# Editorial: Understanding eQTLs and their Association with Spondyloarthropathies

**DOI:** 10.3389/fimmu.2026.1868391

**Published:** 2026-05-13

**Authors:** Matteo Vecellio

**Affiliations:** Centro Ricerche Fondazione Italiana Ricerca in Reumatologia (FIRA), Fondazione Pisana per la Scienza ONLUS, San Giuliano Terme, Italy

**Keywords:** eQTL, genetics, inflammation, microbiome, spondyloarhtopathy

In the recent years, the field of rheumatology has entered in a phase of significant evolution, driven by the integration of immunology, genetics and microbiome research. The four studies presented in this Research Topic collectively illustrate how these complementary perspectives are reshaping our understanding of chronic inflammatory diseases and advancing the prospects for earlier diagnosis, refined risk stratification, and more targeted therapeutic strategies.

In their original article, Van Doorn et al. highlight the therapeutic relevance of immune modulation, showing that rapamycin markedly attenuates HLA−B27–mediated gut inflammation in an experimental model of spondyloarthritis (SpA). By suppressing key pro−inflammatory cytokines, such as IL17A, IL17F and TNF, and altering the composition of the gut microbiome, rapamycin emerges as a compelling candidate for conditions in which HLA−B27–driven immune dysregulation is central. These findings emphasise the importance of mechanistic preclinical models in identifying novel therapeutic avenues for complex immune−mediated disorders, such as axial (ax)SpA. In parallel, Novella-Navarro et al. focus on the metabolic dimension of inflammation. The authors show the importance of serum adipsin and its emerging role as a promising biomarker in both early and established rheumatoid arthritis (RA), with the ability to discriminate RA from axial spondylarthritis and healthy individuals. As the role of adipokines in immune regulation becomes increasingly evident, adipsin may offer new opportunities for early disease detection and improved clinical monitoring.

The genetic architecture of axSpA is reviewed in Awadh et al.’s work. In their manuscript the authors emphasized the growing relevance of expression quantitative trait loci (eQTLs) in SpA pathogenesis. eQTLs might provide a significant functional framework, linking genetic variants to gene−expression changes, for interpreting GWAS findings and then identifying biologically meaningful pathways. Of note, the functional validation of eQTLs represent the most challenging but crucial part to define disease mechanisms and thus inform drug−target discovery and accelerate the transition toward precision medicine.

Consistent with this view, Vecellio et al. address a longstanding goal in clinical rheumatology: the ability to identify individuals at increased risk of developing a chronic inflammatory disease, such as axSpA, based on genetic profiles. The development of sophisticated genetic tools capable of capturing polygenic risk and pathway−level perturbations may ultimately enable earlier identification of high−risk individuals and more effective, personalized intervention strategies. eQTL represent one of the most promising frontiers in the genetic investigation of axSpA. By clarifying how specific genetic variants influence gene expression, eQTL studies provide a functional bridge between inherited risk, dysregulated molecular pathways, and the clinical manifestations of disease.

This approach has the potential not only to deepen our understanding of axSpA pathogenesis, but also to support the development of more precise diagnostic tools and personalized therapeutic strategies. As eQTL maps become increasingly comprehensive and tissue−specific, they may ultimately enable clinicians to identify pathogenic pathways with greater accuracy and tailor interventions to the molecular profile of each patient.

Together, these studies reveal a discipline in transformation. From immune modulation to metabolic biomarkers, from regulatory genetics to predictive risk profiling, rheumatology is moving towards a more integrated and mechanistically informed understanding of disease. This convergence not only deepens our biological understanding of inflammatory rheumatic diseases but also moves the field toward a future in which clinical care is delivered with greater precision and individualization. By integrating insights from immunology, genetics, and microbiome research, these advances collectively pave the way for diagnostic frameworks and therapeutic strategies that are more responsive to the specific molecular and clinical profiles of each patient.

